# Reconstructive Strategies for Abdominal Wall Defects in Patients With Stomas: A Systematic Review of Mesh and Flap Techniques

**DOI:** 10.7759/cureus.116005

**Published:** 2026-09-09

**Authors:** Michael E Chukwu, Chinasa P Okonkwo, Bukola G Adu, Olajire O Salako, Ifeanyichukwu C Ijezie, Emmanuel N Aniaku

**Affiliations:** 1 General Surgery, Brookdale University Hospital Medical Center, New York City, USA; 2 General Surgery, Central Michigan University College of Medicine, Saginaw, USA; 3 General Surgery, Sumy State University, Sumy, UKR; 4 General Surgery, College of Medicine, University of Ibadan, Ibadan, NGA; 5 General Surgery, Independent Research, New York City, USA

**Keywords:** abdominal wall surgery, anterior abdominal wall defect, flap reconstruction, mesh, mesh repair, parastomal hernia, stoma, surgical outcomes

## Abstract

An abdominal wall hernia occurs when there is a defect or weakness in the abdominal wall musculofascial layer. Patients with ostomies are at particular risk of developing parastomal hernia because stoma creation necessarily traverses the abdominal wall, creating a structural weak point. In high-risk patients, including those with prior abdominal surgery, prior mesh infection, recurrent parastomal hernia, or large soft-tissue defects, hernia repair often requires complex reconstruction. Mesh-based repairs reduce hernia recurrence rates; however, limitations persist in settings of infected mesh fields, recurrent parastomal hernias, or extensive soft-tissue defects requiring tissue coverage beyond mesh reinforcement alone.

This systematic review evaluates reconstructive approaches for abdominal wall defects in stoma patients, comparing mesh-only versus mesh with flap repairs, focusing on hernia recurrence, wound healing, and infection outcomes.

Following PRISMA guidelines, comprehensive searches of PubMed, ScienceDirect, and the Cochrane Library were undertaken to identify studies published between January 2015 and July 2025. Boolean operators and Medical Subject Headings (MeSH) terms combining 'stoma,' 'abdominal wall,' and 'reconstruction' yielded 1,947 articles. After duplicate removal, 1,785 unique records were screened. Eligible studies included randomized controlled trials (RCTs), observational studies, and case series with at least five patients, reporting outcomes of mesh with or without flap reconstruction in adult patients undergoing abdominal wall repair in the setting of a stoma, which encompasses parastomal hernia repair, wound breakdown, and complex abdominal wall reconstruction with concomitant stoma management. Exclusions included case reports, reviews, pediatric-only, and abstract-only studies. Screening and data extraction were performed independently by two reviewers, with discrepancies resolved by consensus. Thirteen papers met inclusion criteria. The PRISMA 2020 flow diagram documented study selection.

Included studies comprised retrospective cohorts, case-controlled analyses, and database studies (sample sizes up to 9,000 patients). Patients frequently had malignancy, obesity, prior abdominal surgery, or prior mesh infection as risk factors. Mesh reinforcement, whether prophylactic or used in delayed reconstruction, consistently reduced parastomal hernia incidence. Flap reconstructions, including autologous tissue flaps and component separation, were effective in complex cases involving large defects or recurrent hernias. Techniques such as Stapled Mesh Stoma Reinforcement Technique (SMART) and Stapled Transabdominal Ostomy Reinforcement with Retromuscular Mesh (STORRM) demonstrated feasibility with low recurrence rates in high-risk patients. Both mesh-only and combined mesh-flap approaches demonstrated low complication rates and preserved stoma function, supporting their safety and effectiveness in appropriately selected patients.

Both mesh-only and mesh-plus-flap reconstruction are effective and safe for abdominal wall defect repair in stoma patients. Mesh reinforcement reduces hernia recurrence, while flap techniques offer added benefit in complex cases characterized by large defects, recurrent parastomal hernias, or compromised tissue. Well-powered, prospective comparative studies are needed to establish definitive evidence-based guidelines.

## Introduction and background

Parastomal hernia is the most common long-term complication following stoma creation, with reported incidences of up to 50% [1,2]. In early stages, the condition is often asymptomatic and identified only on routine examination or imaging. When symptomatic, patients experience pain or discomfort around the stoma site. In more severe cases, complications such as incarceration or strangulation may arise, necessitating urgent surgical intervention. While some patients benefit from straightforward mesh repair, those with recurrent parastomal hernias, infected mesh fields, or extensive soft-tissue defects often require complex reconstruction combining mesh and flap techniques.

Recent evidence has shown that prophylactic mesh placement during ostomy creation in high-risk patients can reduce the incidence of parastomal hernia [1-3]. However, prophylactic mesh carries risks including mesh infection, gastrointestinal fistula, and wound-healing complications [4,5]. In clinical practice, reconstruction is therefore typically deferred until symptomatic parastomal hernia develops.

To address limitations of mesh-only repair, particularly in patients with recurrent hernia, infected mesh fields, or large soft-tissue defects, flap-based reconstructions have been employed either alone or in combination with mesh. Techniques such as omental pedicle flaps and autologous tissue flaps provide vascularized coverage, potentially reducing infection risk and improving tissue integration [6-10]. The variation in practice across surgical specialties highlights a lack of consensus on when and how to incorporate flap reconstruction into abdominal wall repair [11]. This heterogeneity, combined with limited comparative data, underscores a critical evidence gap: it remains unclear whether the optimal approach differs between patients undergoing parastomal hernia repair alone versus those requiring complex abdominal wall reconstruction (AWR) with concomitant stoma management.

This systematic review evaluates reconstructive techniques for abdominal wall defects in patients with stomas, comparing outcomes between mesh-only repairs and combined mesh-with-flap reconstruction, across contexts including parastomal hernia repair, wound breakdown management, and complex AWR with concomitant stoma management. Primary outcomes of interest are hernia recurrence, wound healing, and surgical site infection. The aim is to guide clinical decision-making and surgical planning for safe, durable AWR in this population.

## Review

Methodology

Search Strategy

Titles and abstracts were independently screened by two reviewers against predefined eligibility criteria. Full texts of potentially relevant studies were then assessed for inclusion, with discrepancies resolved by consensus or adjudication by a third reviewer.

Inclusion Criteria

The inclusion criteria included randomized controlled trials (RCTs), observational studies (cohort, case-control, cross-sectional), and case series with five or more patients; eligible studies involved adult patients with a stoma undergoing: (i) repair of parastomal hernia (including recurrent cases), (ii) abdominal wall wound breakdown in the setting of a stoma, or (iii) complex AWR with concomitant stoma management. Studies were required to report outcomes of mesh with or without flap reconstruction and to have been published between January 2015 and July 2025.

Exclusion Criteria

The exclusion criteria were case reports, case series with fewer than five patients, systematic reviews, meta-analyses, narrative reviews, expert opinions, abstract-only publications, animal or laboratory studies, pediatric populations, studies not reporting relevant ostomy or abdominal wall outcomes, and studies published prior to January 2015.

The PRISMA 2020 flow diagram, generated using the R Shiny app (R Core Team, Vienna, Austria), was used to document the selection process (Figure 1). Data extraction was performed using a standardized form; two reviewers independently extracted data, with discrepancies resolved through discussion.

**Figure 1:**
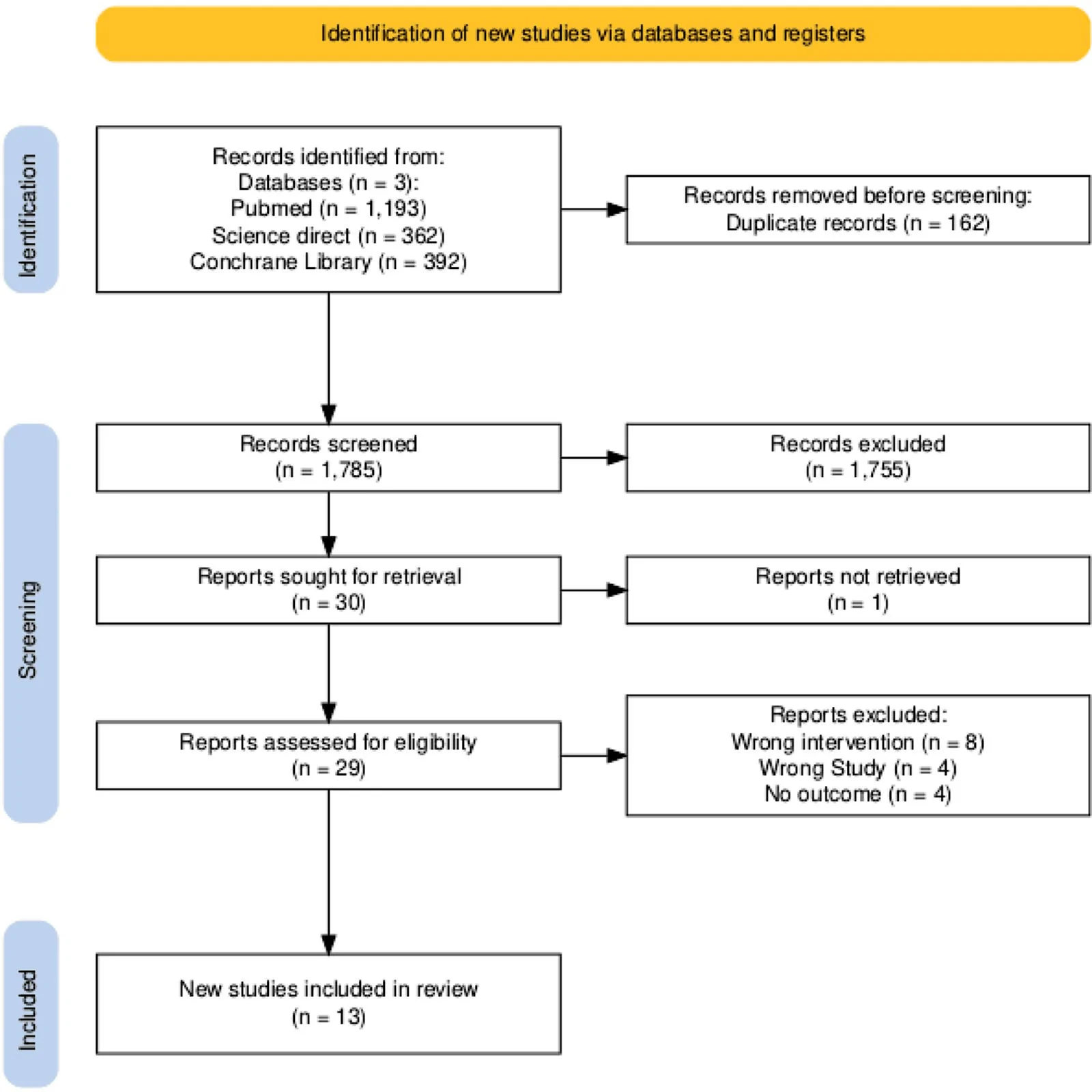
PRISMA flow diagram outlining the study selection process for inclusion in the systematic review of our study

Extracted variables included study characteristics (author, year, country, design, sample size); patient characteristics (age, sex, indication for surgery, comorbidities); intervention details (mesh type, flap technique, combination approach); comparator groups; and outcomes (hernia recurrence, wound healing, surgical site infection, complications, and follow-up duration).

International Prospective Register of Systematic Reviews (PROSPERO) Registration

This systematic review was registered with PROSPERO with registration number CRD420251112031.

Risk of Bias (ROB) and Quality Assessment

The methodological quality of RCTs was assessed using the Cochrane Risk of Bias 2 (RoB 2) tool. Observational studies and case series were evaluated using the Risk Of Bias In Non-randomized Studies of Interventions (ROBINS-I) tool and the Critical Appraisal Skills Programme (CASP) checklists [12-14]. ROB assessments were performed independently by two reviewers, with disagreements resolved by consensus. Results are summarized in Table 1.

**Table 1:** Summary of risk-of-bias assessments using the ROBINS-I tool for nonrandomized studies and the ROB 2 tool for randomized controlled trials (RCTs) ROBINS-I: Risk Of Bias In Non-randomized Studies of Interventions; ROB 2: Risk of Bias 2

Study (Year)	Study Design	ROB Tool	Confounding	Selection	Classification	Deviations	Missing Data	Outcome Measurement	Selective Reporting	Overall ROB
Mericli et al. (2017) [4]	Retrospective	ROBINS-I	Serious	Moderate	Low	Low	Moderate	Moderate	Low	Serious
Wong et al. (2020) [3]	Retrospective	ROBINS-I	Serious	Moderate	Low	Low	Low	Moderate	Low	Serious
Maskal et al. (2024) [15]	Retrospective	ROBINS-I	Serious	Moderate	Low	Low	Low	Low	Low	Serious
Wallace et al. (2023) [16]	Prospective	ROBINS-I	Moderate	Low	Low	Low	Low	Low	Low	Moderate
Chegireddy et al. (2021) [8]	Retrospective	ROBINS-I	Serious	Serious	Low	Low	Moderate	Moderate	Low	Serious
Shmelev et al. (2023) [9]	Retrospective	ROBINS-I	Moderate	Moderate	Low	Low	Low	Low	Low	Moderate
Sweitzer et al. (2024) [17]	Retrospective	ROBINS-I	Serious	Moderate	Low	Low	Moderate	Moderate	Low	Serious
Majumder et al. (2018) [18]	Prospective	ROBINS-I	Moderate	Low	Low	Low	Moderate	Low	Low	Moderate
Ozben et al. (2016) [6]	Retrospective	ROBINS-I	Serious	Moderate	Low	Low	Low	Moderate	Low	Serious
Lake et al. (2024) [10]	Prospective	ROBINS-I	Moderate	Low	Low	Low	Moderate	Low	Low	Moderate
Vierimaa et al. (2015) [1]	RCT	ROB 2.0	Low	Low	Low	Low	Low	Low	Low	Low
Williams et al. (2015) [2]	RCT	ROB 2.0	Some concerns	Low	Low	Low	Moderate	Low	Low	Some concerns
Giordano et al. (2018) [5]	Retrospective	ROBINS-I	Serious	Moderate	Low	Low	Moderate	Low	Low	Serious

The ROB assessment across the included studies highlights important considerations regarding the trustworthiness of the current evidence base. Most retrospective analyses demonstrated some concerns related to confounding, selection bias, and outcome assessment, leading to reduced confidence in their internal validity. Prospective studies generally performed better, with moderate risks, reflecting more robust methodological design but still some limitations in missing data and selective reporting. The RCTs presented the lowest ROB; this, therefore, added valuable strength to the evidence hierarchy. Taken all these together, the findings suggest that while the collective evidence is informative, the overall certainty remains moderate, warranting cautious interpretation of this data and emphasizing the overall need for well‑designed randomized investigations in the future.

Results

The extracted data included several studies examining reconstructive approaches to abdominal wall defects in patients with stomas, focusing on both flap and mesh techniques. The studies varied in design, encompassing retrospective cohort analyses, case-controlled pilot studies, and prospective database analyses, with sample sizes ranging from small pilot cohorts to over 9,000 patients [3,4,9,15,16]. Study characteristics, including design, population, interventions, and outcomes for each included study, are summarized in Table 2.

**Table 2:** Characteristics of the articles included in the review AWR: abdominal wall reconstruction; SSO: surgical site occurrence; SSI: surgical site infection; ADM: acellular dermal matrix; SBO: small bowel obstruction; OPF: omental pedicle flap; RCT: randomized controlled trial

Authors	Country	Year	Study Design	Sample Size	Male	Female	Age	Comparator	Intervention	Outcome
Mericli et al. [4]	United States	2017	Retrospective study	499	242	257	Mean 58.2 ± 12.3 years (AWR) vs. 61.4 ± 12.3 years (AWR+O); after matching: 58 vs. 60 years	AWR without ostomy-associated hernia repair	AWR with concomitant ostomy-associated hernia repair (AWR+O), mostly parastomal hernia; techniques included keyhole and Sugarbaker with ADM	Primary: No significant difference in ventral hernia recurrence. Secondary: Higher complications in AWR+O: SSO, SSI, wound dehiscence, longer hospital stay, and more reoperations.
Wong et al. [3]	Australia	2020	Retrospective study	935 procedures, 645 patients	358	287	Median 65 years (range 20-96)	Standard abdominal wall closure without mesh	Prophylactic polypropylene onlay mesh placement at time of midline laparotomy or ileostomy reversal	Incisional hernia complications: Slightly higher seroma and chronic pain in mesh group; infection
Maskal et al. [15]	United States	2024	Retrospective study	438	Not explicitly reported	Not explicitly reported	Median age ~60 years (no significant difference between groups)	Single-stage AWR - hernia repair with permanent mesh during contaminated or clean-contaminated abdominal surgery	Delayed AWR - initial contaminated abdominal surgery performed without mesh; later definitive repair with permanent synthetic mesh	No significant difference in wound morbidity, reoperation, or recurrence between delayed and single-stage approaches
Wallace et al. [16]	United Kingdom	2023	Prospective study	62	33	29	58 ± (35-77)	Complex AWR with intestinal surgery	Complex AWR	Hernia recurrence, SSI, mesh infection, operative time
Chegireddy et al. [8]	United States	2021	Retrospective study	22	9	13	66.7	None	Three-dimensional silo technique using biologic mesh	Recurrence: 3/22 (13.6%); complications: three infections, one seroma, two healing delays, six readmissions (three SBOs)
Shmelev et al. [9]	United States	2023	Retrospective study	9206	9206	0	58.99	None	Mesh	Infection, re-operation
Sweitzer et al. [17]	United States	2024	Retrospective study	254	117	137	57.5 ± 11.8	Non-reinforced mesh placement	Reinforced mesh (66) vs non-reinforced mesh (188)	90-day postoperative complications and hernia recurrence
Ozben et al. [6]	United States	2016	Retrospective study	468	326	117	59.4 ± 12	Non-OPF	65 in OPF group, 403 in non-OPF	Primary: anastomotic leak (AL); secondary: SSI
Lake et al. [10]	United States	2024	Prospective observational cohort study	28	16	12	58.5 (±16.3)	None	Placement of a reinforced tissue matrix (RTM) used to reinforce the abdominal wall at hernia repair and stoma takedown; deployed via laparoscopic, robotic, or open surgical approaches with either sublay (82.1%) or intraperitoneal onlay (IPOM; 17.9%) positioning	Hernia recurrence - 0% recurrence at both one-month and one-year follow-up
Vierimaa et al. [1]	Finland	2015	Prospective RCT	70	43	27	67	Standard laparoscopic end colostomy formation without mesh	Prophylactic laparoscopic placement of a dual-component intraperitoneal onlay mesh around the stoma	Primary: Parastomal hernia incidence (clinical + radiological) at 12 months. No significant difference in stoma-related morbidity
Williams et al. [2]	United Kingdom	2015	RCT	22	6	16	49 ± 16	Stoma resiting without mesh reinforcement	Stapled Mesh stomA Reinforcement Technique (SMART)	Recurrence
Giordano et al. [5]	United States	2018	Retrospective study	452	220	232	58 ± 12.3	None	AWR with acellular dermal matrix	Primary: unplanned readmission within 30days of initial surgery. Secondary: recurrence at follow-up
Majumder et al. [18]	United States	2018	Prospective study	12	8	4	64 ± 14	None	Stapled Transabdominal Ostomy Reinforcement with retromuscular mesh	Recurrence, surgical site infection

Patient Characteristics and Study Population

Patients included in these studies were undergoing stoma formation for a variety of indications, including colorectal malignancies, elective resections, and emergency procedures. Specific patient risk factors, such as previous abdominal surgery or high body mass index, were considered in several studies when evaluating hernia risk and outcomes [3,4,15,16]. A retrospective cohort study of about 254 patients conducted in 2024 highlighted that prior mesh infection elevated recurrence risk and a BMI > 35 increased complications in patients [1]. These studies consistently highlighted that high-risk patients were the primary target for prophylactic or reconstructive interventions.

Reconstructive Techniques

Reconstructive approaches varied across the studies. Mesh reinforcement was employed in several cohorts, either prophylactically during stoma formation or as part of delayed AWR [3,9,15,16]. Flap-based techniques, including autologous tissue flaps and component separation procedures, were also reported, with some studies comparing these to traditional mesh repairs [7,10,17,19]. Several studies specifically compared autologous flap loops to conventional loop ileostomies, evaluating outcomes such as hernia recurrence, quality of stoma, and complication rates [10,18]. One study found no association between open primary flap (OPF) use and incidence of active leak [6]. Another study reported that the size of the pedicle innervating the vastus lateralis and anterolateral thigh (PIVA) flap did not significantly affect quality of life or donor-site morbidity [7].

The Stapled Mesh Stoma Reinforcement Technique (SMART) was reported in a case-controlled pilot study with 22 patients, demonstrating its application in high-risk stoma formation and highlighting both its feasibility and potential effectiveness [2]. The parastomal hernia repair with Stapled Transabdominal Ostomy Reinforcement with Retromuscular Mesh (STORRM) technique is particularly advantageous in parastomal hernia repair, especially when tissue planes are disrupted or when the stoma has a small diameter, as is often the case with an ileal conduit [19]. The technique involved simultaneous stoma creation with mesh reinforcement, aiming to reduce fascial defects and prevent parastomal hernia.

Primary Outcomes: Hernia Recurrence

Across the studies, hernia recurrence was the most commonly reported primary outcome. Mesh-based interventions consistently showed reductions in hernia incidence compared to conventional stoma formation without reinforcement [3,9,15,16]. The SMART technique was associated with lower recurrence rates in high-risk patients, with no reported early hernias during follow-up periods documented in the pilot study [2]. Flap-based reconstructions, including autologous flap loops, were also effective in preventing hernia formation, with one study noting reduced hernia incidence in the flap group compared to traditional loop ileostomies [19].

Secondary Outcomes: Safety and Postoperative Complications

The studies reported secondary outcomes such as wound complications, operative feasibility, and early postoperative morbidity. Overall, both mesh and flap techniques were reported as safe, with minimal major complications [1-4,7-10,15-17,19]. The use of mesh and flap techniques was associated with minimal complications, none of which necessitated additional intervention or impaired stoma function [1,3,9]. Specific studies emphasized procedure feasibility, noting that interventions like the SMART technique and autologous flap reconstruction could be integrated into standard surgical workflows without intraoperative issues [2,10,19].

Quality of stoma and patient-reported outcomes were specifically highlighted in studies involving autologous flap techniques, indicating acceptable stoma function and satisfactory postoperative outcomes [7,8,15,19].

Comparative Observations and Trends

Comparisons across studies suggested that mesh reinforcement, particularly when applied prophylactically, reduced the risk of parastomal hernia, while flap-based reconstructions provided an effective alternative for patients with complex abdominal wall defects [2,3,15,16,19]. Both approaches demonstrated low rates of serious complications, supporting their safety. The pilot and case-controlled studies emphasized the value of techniques that reinforce the stoma site, particularly in patients at elevated risk of hernia formation [2,19].

Summary of Findings

In summary, the extracted data indicate that both mesh-based and flap-based reconstruction approaches are effective for preventing hernia recurrence in patients undergoing stoma creation. The SMART procedure, autologous flap techniques, and traditional mesh reinforcement demonstrated reductions in parastomal hernia rates, while maintaining a favorable safety profile and procedural feasibility [2-4,7,9,10,15,16,19]. These findings support the use of prophylactic reinforcement techniques in high-risk populations and provide a foundation for further research into optimizing AWR strategies in stoma patients.

Discussion

This systematic review evaluated reconstructive outcomes in patients with stomas requiring abdominal wall repair, encompassing three overlapping clinical scenarios: parastomal hernia repair, wound breakdown management, and complex AWR with concomitant stoma management. The findings consistently support the effectiveness and safety of both mesh-only and mesh-plus-flap approaches, while highlighting the role of clinical complexity in guiding technique selection.

Across the included studies, mesh reinforcement, whether applied prophylactically at stoma creation or as part of delayed reconstruction, was the most consistently reported intervention and was associated with reductions in parastomal hernia incidence [3,9,15,16]. The two RCTs [1,2] provide the strongest evidence in this review. Vierimaa et al. (2015) demonstrated that prophylactic intraperitoneal onlay mesh placement at end colostomy formation did not increase stoma-related morbidity, while Williams et al. (2015) found that the SMART technique was feasible and associated with no early hernia recurrence in a high-risk population. These findings reinforce the value of reinforcement at the time of stoma creation in appropriately selected patients.

Flap-based reconstruction provided added benefit in the subset of patients with more complex presentations, including those with recurrent parastomal hernias, large soft-tissue defects, or infected operative fields. Mericli et al. (2017) reported that concomitant ostomy-associated hernia repair during AWR+O did not result in significantly higher ventral hernia recurrence compared to AWR alone, though it was associated with higher wound morbidity [4]. Wallace et al. (2023) demonstrated that gastrointestinal procedures and anastomoses can be safely performed during complex AWR, suggesting that combined approaches are surgically feasible [16]. The use of biologic mesh in a three-dimensional silo configuration (Chegireddy et al., 2021) achieved a recurrence rate of 13.6% with a manageable complication profile in a small cohort [8].

Autologous flap techniques, including reinforced tissue matrix, omental pedicle flaps, and the PIVA flap, contributed vascularized coverage, potentially reducing infection risk and improving durability in contaminated or extensively scarred fields [6,7,10]. Lake et al. (2024) reported 0% hernia recurrence at one year using a reinforced tissue matrix at hernia repair and stoma takedown [10], suggesting that tissue-integrated biomaterials may offer durable outcomes. Similarly, Majumder et al. (2018) found the STORRM technique to be feasible for parastomal hernia repair in cases where tissue planes are disrupted [18].

Taken together, these findings suggest that the choice of reconstructive strategy should be individualized based on defect complexity, the presence of recurrent hernia or prior mesh infection, soft-tissue availability, and patient comorbidities. Mesh repair remains the primary approach for most cases; combined mesh and flap reconstruction should be reserved for patients with large defects, recurrent hernias, contaminated fields, or compromised soft tissue where mesh alone is unlikely to be sufficient.

Several limitations of the included literature warrant consideration. The majority of studies were retrospective single-center cohorts with significant heterogeneity in surgical techniques, mesh types, flap selection, and outcome definitions. Follow-up durations were variable and often short. Only two RCTs were identified, both of limited sample size. These factors precluded meta-analysis and limit the strength of comparative conclusions. Well-powered, prospective comparative studies are needed to establish clear evidence-based guidelines for this population.

Limitations

This systematic review has several limitations. First, the majority of included studies were observational in design, with only two RCTs identified, limiting the ability to draw strong causal inferences. Second, significant heterogeneity existed across studies in surgical techniques, flap selection, mesh types, defect size, and outcome definitions, precluding formal meta-analysis and limiting direct comparisons. Third, the review was restricted to English-language publications available in full text, potentially introducing language and publication bias. Fourth, access to certain databases (e.g., Embase) was limited, which may have resulted in omission of relevant studies. Fifth, follow-up durations were variable and often short, and reporting of functional and patient-reported outcomes was inconsistent. Sixth, the 10-year search window (2015-2025) was chosen to reflect the current state of surgical practice; while this improves contemporary relevance, it necessarily excludes foundational earlier evidence. Finally, evolving surgical techniques and perioperative practices over the study period may affect the generalizability of pooled findings.

## Conclusions

This systematic review of 13 studies demonstrates that both mesh-only reconstruction and combined mesh and flap reconstruction are effective options for managing abdominal wall defects in stoma patients, with safety profiles that support their use in appropriately selected individuals. Mesh reinforcement, whether applied prophylactically at the time of stoma formation or as part of a delayed repair strategy, consistently reduced parastomal hernia incidence without significant increases in stoma-related morbidity, as demonstrated across multiple included studies. The benefit of combined mesh and flap reconstruction was particularly evident in complex clinical scenarios, including those involving recurrent hernias, infected mesh fields, large soft-tissue defects, or disrupted tissue planes. In these settings, the combined approach demonstrated added durability and acceptable wound outcomes. Of note, a 0% hernia recurrence rate at one year was reported with a reinforced tissue matrix in one prospective cohort, and the STORRM technique demonstrated feasibility in cases with small stoma diameters or disrupted tissue planes.

Emerging techniques such as SMART and STORRM show promise in high-risk populations, though evidence at this time remains limited to small cohorts and pilot studies. Across all included studies, no single technique emerged as universally superior across all patient profiles. High-quality RCTs with standardized outcome definitions and adequate follow-up duration are urgently needed to establish evidence-based guidelines for this patient population. Until such evidence becomes available, surgical decision-making should remain individualized, guided by patient comorbidities, defect characteristics, and surgeon expertise.
